# Extraction of microalgae derived lipids with supercritical carbon dioxide in an industrial relevant pilot plant

**DOI:** 10.1007/s00449-017-1755-5

**Published:** 2017-03-15

**Authors:** Jan Lorenzen, Nadine Igl, Marlene Tippelt, Andrea Stege, Farah Qoura, Ulrich Sohling, Thomas Brück

**Affiliations:** 1 Department of Chemistry, Technical University of Munich, Lichtenbergstrasse 4, 85748 Garching, Germany; 2 Hopfenveredlung St. Johann GmbH & Co. KG, Auenstr. 18-20, 85283 Wolnzach, Germany; 3 Clariant Produkte (Deutschland) GmbH, Ostenrieder Str. 15, 85368 Moosburg, Germany

**Keywords:** Supercritical carbon dioxide extraction, Microalgae, *Scenedesmus*, Lipids, Bentonite

## Abstract

Microalgae are capable of producing up to 70% w/w triglycerides with respect to their dry cell weight. Since microalgae utilize the greenhouse gas CO_2_, they can be cultivated on marginal lands and grow up to ten times faster than terrestrial plants, the generation of algae oils is a promising option for the development of sustainable bioprocesses, that are of interest for the chemical lubricant, cosmetic and food industry. For the first time we have carried out the optimization of supercritical carbon dioxide (SCCO_2_) mediated lipid extraction from biomass of the microalgae *Scenedesmus obliquus* and *Scenedesmus obtusiusculus* under industrrially relevant conditions. All experiments were carried out in an industrial pilot plant setting, according to current ATEX directives, with batch sizes up to 1.3 kg. Different combinations of pressure (7–80 MPa), temperature (20–200 °C) and CO_2_ to biomass ratio (20–200) have been tested on the dried biomass. The most efficient conditions were found to be 12 MPa pressure, a temperature of 20 °C and a CO_2_ to biomass ratio of 100, resulting in a high extraction efficiency of up to 92%. Since the optimized CO_2_ extraction still yields a crude triglyceride product that contains various algae derived contaminants, such as chlorophyll and carotenoids, a very effective and scalable purification procedure, based on cost efficient bentonite based adsorbers, was devised. In addition to the sequential extraction and purification procedure, we present a consolidated online-bleaching procedure for algae derived oils that is realized within the supercritical CO_2_ extraction plant.

## Introduction

Recently, governmental CO_2_ emission regulations and an increased awareness of sustainability drive the development of renewable feedstocks based industrial processes. Hence, many different renewable feedstocks have been tested in the last decades, like organic waste, microbe-derived lipids or different types of plant seeds. With respect to sustainability and lipid productivity, microalgae are deemed to be one of the most relevant feedstocks for lipid type chemical products [1–3]. Conservative estimations postulate that about 72.000 algae species exist [4], most of them represented by microalgae. Compared to other renewable feedstocks for bio-lipid production, like rapeseed or soybeans, microalgae show several beneficial characteristics. In contrast to vascular plants, microalgae exhibit high growth rates at low area consumption. Lipid contents higher than 50% are reported for various species and can be controlled by the composition of the cultivation medium. A nitrogen starvation, for example, leads to a significant increase in lipid-storage in different microalgae [5–7]. In addition to these arguments, one of the most important advantage of microalgae is the lack of competition with agricultural activities. In this context, it is also important to mention that many lipid-producing microalgae can be cultivated in brackish or salt water, which secures valuable freshwater resources for human activity.

One of the main obstacles to overcome in the usage of microalgae derived lipids is to find a method for lipid extraction that is efficient, economically relevant and environmentally friendly. One of the most common lipid extraction methodologies is unspecific organic solvent extraction according to the work of Bligh and Dyer [8]. Other methods combine solvent and/ or enzyme assisted techniques that are either highly toxic (n-hexane, methanol) or energetically inefficient [9]. By contrast, lipid extraction with supercritical fluids, especially supercritical carbon dioxide, has recently risen as a powerful industrial tool for environmentally friendly lipid recovery from biomass. Recently, many reports have discussed the extraction of microalgae lipids with supercritical carbon dioxide in small pilot plants, in the majority of the cases with a polar co-solvent (e.g., ethanol or water [10, 11]), an approach that cannot be transformed to industrial scale due to legislative safety regulations.

In this study, we optimized the performance of an industrially relevant supercritical carbon dioxide extraction process for the recovery of lipids from dried microalgae biomass for the first time. The extraction of microalgae lipids with supercritical carbon dioxide was performed in a pilot plant according to European industrial standards for large scale supercritical fluid extractions (ATEX directives). Furthermore, we present a subsequent purification procedure for the crude microalgae lipid extracts, to provide a lipid fraction that is applicable for bio-lubricant/bio-fuel and cosmetic applications.

##  Materials and methods

### Strains and cultivation

Two different batches of algae biomass have been analyzed. The first batch was provided by Hochschule Anhalt, a heterologous culture consisting of 85–90% *Scenedesmus obliquus*, low percentages of *Chlorella vulgaris* and *Chlorella kessleri* as well as traces of *Chlorella vacuolatus*. The algae were cultivated under non-limiting conditions with natural sunlight illumination. The second batch analyzed was an unialgal culture of *Scenedesmus obtusiusculus*, cultivated in BG-11 medium under non-limiting conditions, using LED-assisted natural sunlight illumination. After the cultivation period, the cells were harvested from the cultivation broth, cracked by high pressure homogenization and lyophilized.

### Conventional solvent extraction

The conventional extraction of lipids from microalgae biomass was performed according to Bligh and Dyer [8], with hexane as single solvent for 8 h.

### Supercritical carbon dioxide (SCCO_2_) extraction


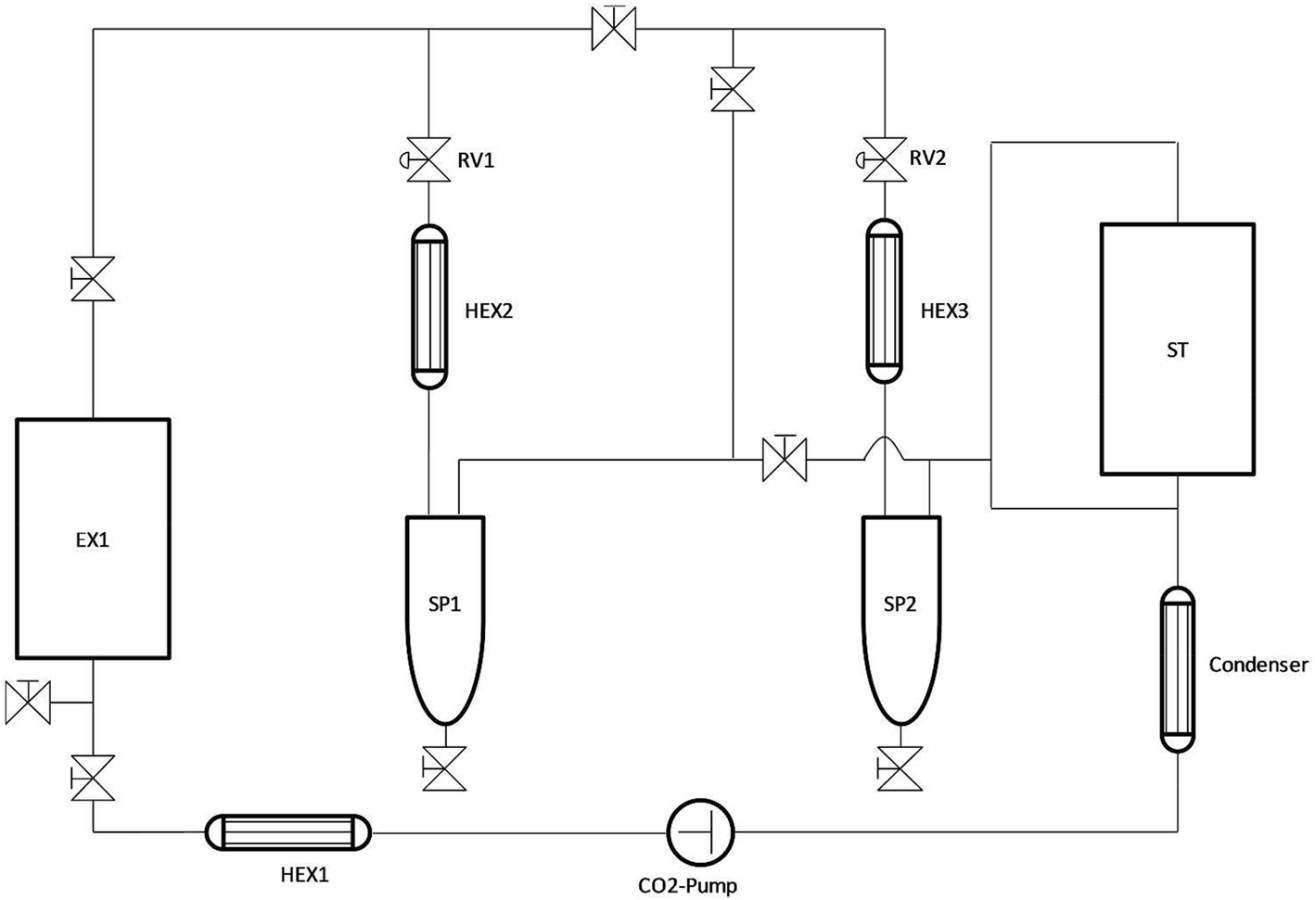


Liquid CO_2_ from the storage tank (ST) is pressurized by a CO_2_-pump and heated to the extraction temperature by a heat exchanger (HEX 1). Subsequently, the CO_2_ flows in the extraction vessel (EX1) containing the biomass. The CO_2_/extract mixture is separated by controlled pressure reduction under simultaneous warming by heat exchangers (HEX2, HEX3) into a CO_2_ vapour and an extract phase (separators SP1, SP2). The extracts are removed from the process while the gaseous CO_2_ remains in the process. Afterwards, the CO_2_ is liquefied by a condenser and recirculated.

All extractions were performed with sample sizes from 650 to 1300 g of lyophilized algae biomass. In terms of the high amount of biomass required for the extraction in the pilot plant, not all experiments could be carried out in duplicates. The online bentonite bleaching was only tested on the biomass of the unialgal *S. obtusiusculus* culture. The tests were focused on this strain, because it showed the most promising process parameters in terms of cultivation stability and biomass yields and therefore became the leading strain in the Advanced Biomass Value (ABV) project.

### Purification and analysis of microalgae extracts

#### Lipid analysis

The direct transesterification of the algae derived lipids was performed according to a modified protocol of Griffiths et al. [12] with the following modifications: replacement of the C17-TAG by a C12-TAG, replacement of BF3 methanol by a HCL-methanol solution, and the C19-ME was omitted. Subsequently, the resulting fatty acid methyl ester (FAME) extract was injected into a Thermo Scientific™ TRACE™ Ultra Gas Chromatograph coupled to a Thermo DSQ™ II mass spectrometer and the Triplus™ Autosampler injector. Column: Stabilwax^®^ fused silica capillary (30 m × 0,25 mm, film thickness 0.25 μm). (Program: initial column temperature 50 °C, increasing (4 °C/min) up to a final temperature of 250 °C. Carrier gas: hydrogen, flow rate 3.5 mL/min.) Peaks were identified by comparison to a marine oil standard (Restek) or by specific molecular masses detected.

#### Purification of crude micro algae extracts

The lipids were purified with an adsorbent based on montmorillonite. In a column with n-hexane 20 g Tonsil 510^®^ from Clariant Produkte (Deutschland) GmbH were filled and given 10 min to swell in the solvent. 2–3 g algae oil were applied to the column and given time to sink completely into the adsorbent. The elution was done with n-hexane until 150 ml eluate was collected. After removing the solvent with a rotary evaporator and drying the oil at 110 °C for 1 h, the oil was analyzed via inductively coupled plasma optical emission spectroscopy (ICP-OES) with a Perkin Elmer Optima 3300 DV, analog to DIN EN ISO 11885. The samples were dissolved in kerosene.

## Results

###  Effect of pressure and temperature on extraction yield

The major points of regulation during the process of SCCO_2_ extraction are changes in extraction temperature and the applied pressure. For the first experiments, temperature and pressure profiles established in the pilot plant from astaxanthin extraction were tested on the lyophilized biomass of the mixed *Scenedesmus* culture (Table 1).

**Table 1:** Adapted temperature and pressure profiles of astaxanthin extractions, applied to mixed, lyophilized Scenedesmus biomass and the corresponding extraction yields after SCCO_2_ extraction and soxhlet extraction of the spent material

Extraction pressure (MPa)	Extraction temp. (°C)	CO_2_: biomass ratio	Extraction yield (% w/w)	Soxhlet yields of spent material (% w/w)
30	50	100	6.9	1.2
50	60	100	6.7	0.5
60	60	200	5.8	0.9
80	80	100	7.6	0.2

Extraction time 540 min

The different temperature and pressure profiles resulted in comparable quantities of extracted lipids, but showed large differences in the quality of the extracts. The extracts obtained from the profiles with lower temperature and pressure were unclear, greenish-brownish, viscous liquids, whereas the extracts gained from the profiles with high temperature and pressure appeared as very dark brownish, almost black and extremely viscous substance. For most of the downstream applications of the extracted algae lipid fractions, a clear and homogeneous extract is required. In terms of developing an economically and ecologically balanced extraction process and to obtain a more clear and homogeneous extract, the following experiments were carried out under milder conditions (Table 2).

**Table 2:** Different extraction profiles applied to mixed, lyophilized Scenedesmus biomass and the corresponding extraction yields after SCCO_2_ extraction and soxhlet extraction of the spent material

Profile	Extraction pressure (MPa)	Extraction temp. (°C)	CO_2_: biomass ratio	Extraction yield (% w/w)	Soxhlet yields of spent material (% w/w)
1	7	20	20	6.5	1.0
2	7	20	100	6.6	2.0
3	12	20	20	6.6	1.8
4	12	20	100	8.3	2.1
	12	20	100	7	2.8
5	15	20	100	6.6	3.0
	15	20	100	6.5	2.8

Extraction time 540 min

The milder extraction conditions applied to the biomass samples resulted in lipid yields between 6.5 and 8.3% (w/w), suggesting that there is no statistically relevant distinction in the extraction efficiency compared to the extraction profiles applied before (Table 1). Although equal quantities were obtained, the quality of the extracts strongly improved. The obtained extracts are clear, homogenous liquids with slightly different colors. The extracts from the profiles 1 and 3 had a greenish color, while the extracts from the other three profiles showed a more brownish to orange color.

The extraction profile 4 showed the best results in this experimental set-up, with respect to the extraction effency and sustainability, and was employed for subsequent extractions.

**Fig. 1:**
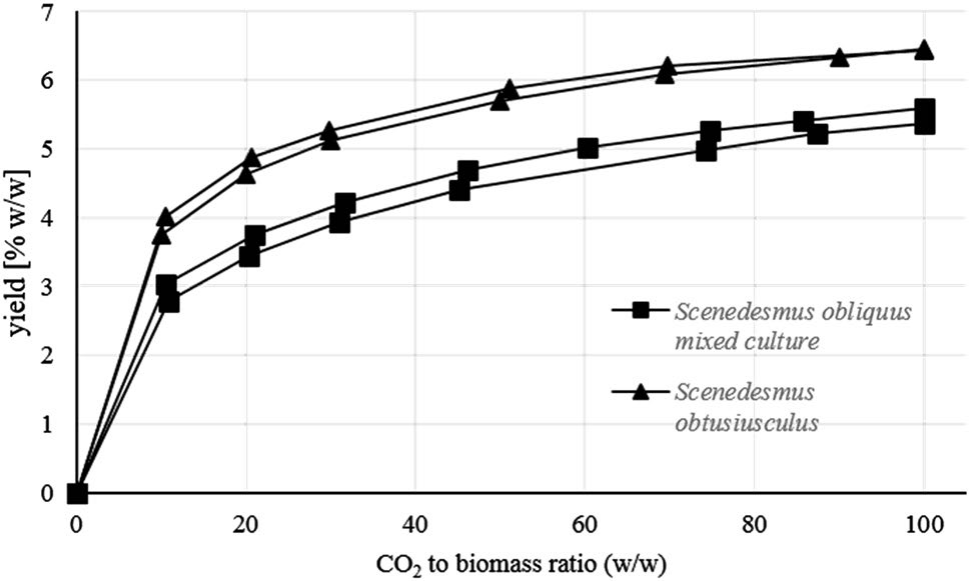
Algae lipid extraction yields from two samples of a mixed *Scenedesmus obliquus* culture (*square* markers) and from two samples of an unialgal *Scenedesmus obtusiusculus* culture (*triangle markers*), expressed as a function of increasing CO_2_ to biomass ratio. Extraction time 840 min

As a proof of concept the extraction profile was applied to an unialgal *S. obtusiusculus* culture (Table 3) and was used to monitor the extraction efficiency (Fig. 1).

**Table 3:** Extraction parameters applied to *Scenedesmus obtusiusculus* biomass (unialgal culture) and the corresponding extraction yields after SCCO_2_ extraction and soxhlet extraction of the spent material (in duplicate)

Extraction pressure (MPa)	Extraction temp. (°C)	CO_2_: biomass ratio	Extraction yield (% w/w)	Soxhlet yields of spent material (% w/w)
12	20	100	6.4	0.5
12	20	100	6.4	0.9

Extraction time 540 min

The transferability of the extraction profile to other microalgae species within the *Scenedesmus* family is depicted in Table 3. Both quantity and quality of the extracts were comparable to those of the mixed *S. obliquus* culture extracted previously.

The extraction curves in Fig. 1 depict the extraction yields (in % w/w) of four different samples, two samples of a mixed *S. obliquus* culture (square markers) and two samples of an unialgal *S. obtusiusculus* culture (triangle markers), as a function of the CO_2_ to biomass ratio. The four extraction curves follow a sigmoidal function and are almost reaching a plateau of maximum lipid yield at a ratio of 100 (CO_2_/biomass), matching the results shown in Table 1. In addition, the technical duplicates of the extraction curves demonstrate that the extraction method is reproducible.

###  Microalgae extract analysis

#### Fatty acid composition of extracts obtained from different extraction profiles

The analysis of the fatty acid (FA) composition of the microalgae extracts (Table 4) showed no significant difference for the extraction profiles tested. The major compounds in these extracts are linolenic acid (C18:3), hexadecatetraenoic acid (C16:4), linoleic acid (C18:2), oleic acid (C18:1) and palmitic acid (C16:0). The FA profile of the unialgal culture of *S. obtusiusculus* was similar to the one from the mixed *S. obliquus* culture in its major components, but the percentage of FAs identified from the total lipid extract is reduced by 20–30%. The obtained FA profiles accord with previously published profiles.

**Table 4:** Major fatty acid (FA) components in microalgae extracts from different extraction profiles, expressed as percentage of the total lipid extract (mean ± standard deviation)

	*S. obliquus*							*S. obtusiusculus*
Profile	1	2	3	4		5		4
Pressure (MPa)	7	7	12	12	12	15	15	12
CO_2_: biomass ratio	20	100	20	100	100	100	100	100
C14	0.29 ± 0.01	0.27 ± 0.02	0.27 ± 0.01	0.29 ± 0.01	0.30 ± 0.01	0.29 ± 0.02	0.29 ± 0.02	0.16 ± 0.00
C16	6.30 ± 0.19	6.38 ± 0.09	6.46 ± 0.22	6.01 ± 0.05	5.68 ± 0.03	6.56 ± 0.09	6.07 ± 0.02	3.25 ± 0.05
C16:1 cis ω7	0.72 ± 0.02	0.72 ± 0.01	0.74 ± 0.03	0.74 ± 0.03	0.78 ± 0.02	0.77 ± 0.01	0.77 ± 0.01	0.45 ± 0.01
C16:1 ω9	1.40 ± 0.06	1.83 ± 0.02	1.53 ± 0.05	2.16 ± 0.03	2.03 ± 0.02	2.01 ± 0.00	1.89 ± 0.01	0.41 ± 0.03
C16:2 ω6	1.24 ± 0.04	1.31 ± 0.02	1.27 ± 0.04	1.43 ± 0.02	1.48 ± 0.07	1.44 ± 0.02	1.41 ± 0.01	0.71 ± 0.01
C16:3 ω3	2.31 ± 0.09	2.54 ± 0.02	2.28 ± 0.04	2.77 ± 0.03	2.77 ± 0.03	2.72 ± 0.02	2.70 ± 0.01	1.13 ± 0.02
C16:4 ω3	9.81 ± 0.33	11.93 ± 0.02	9.56 ± 0.55	13.25 ± 0.09	13.28 ± 0.44	12.66 ± 0.23	12.53 ± 0.16	3.91 ± 0.07
C18	0.13 ± 0.00	0.15 ± 0.00	0.14 ± 0.00	0.16 ± 0.00	0.15 ± 0.01	0.15 ± 0.00	0.14 ± 0.00	0.17 ± 0.01
C18:1 ω9	6.59 ± 0.14	7.02 ± 0.08	6.93 ± 0.23	7.43 ± 0.08	7.45 ± 0.07	7.24 ± 0.09	7.25 ± 0.07	4.09 ± 0.03
C18:1 ω7	1.20 ± 0.02	1.28 ± 0.01	1.25 ± 0.04	1.38 ± 0.04	1.38 ± 0.01	1.34 ± 0.02	1.34 ± 0.03	0.79 ± 0.00
C18:2 ω6	9.04 ± 0.18	9.75 ± 0.11	9.18 ± 0.05	10.20 ± 0.07	10.21 ± 0.08	9.94 ± 0.15	9.94 ± 0.10	6.93 ± 0.07
C18:3 ω3	24.78 ± 0.44	27.44 ± 0.24	24.75 ± 0.59	28.12 ± 0.16	28.44 ± 0.28	27.69 ± 0.35	27.75 ± 0.07	20.62 ± 0.29
C18:4 ω3	2.26 ± 0.09	2.81 ± 0.02	2.25 ± 0.12	3.02 ± 0.03	3.07 ± 0.01	2.96 ± 0.05	2.95 ± 0.01	0.88v ± 0.02
C20	ND	ND	ND	0.03 ± 0.04	0.02 ± 0.00	0.02 ± 0.00	ND	ND
C20:1 ω9	0.05 ± 0.04	0.10 ± 0.01	0.05 ± 0.04	0.08 ± 0.00	0.08 ± 0.01	0.08 ± 0.01	0.10 ± 0.01	ND
FA yield	65.72 ± 1.58	73.53 ± 0.37	66.66 ± 0.83	77.07 ± 0.48	77.09 ± 0.87	75.87 ± 0.59	75.11 ± 0.09	43.52 ± 0.52

All extractions were carried out at 20 °C

*FA yield* percentage of FAs from total lipids, *ND* not detectable

#### Processing of microalgae oils with bentonites

The microalgae lipid fractions obtained from the mild extraction profiles show a higher quality then the ones obtained under harsh extraction conditions. Anyway the coloring of the extracts implements a relatively high amount of substances like chlorophylls and carotenoids. As these substances could be problematic for any downstream usage of the microalgae oils, e.g., in technical applications, the lipid fractions were processed with the bentonite Tonsil 510^®^.

The results of a single treatment of microalgae oils with the bentonite are shown in Table 5. After the processing the lipid fractions appear as a clear, liquid oil, suggesting that the coloring substances like chlorophyll and carotenoids have been completely removed from the extracts.

**Table 5:**
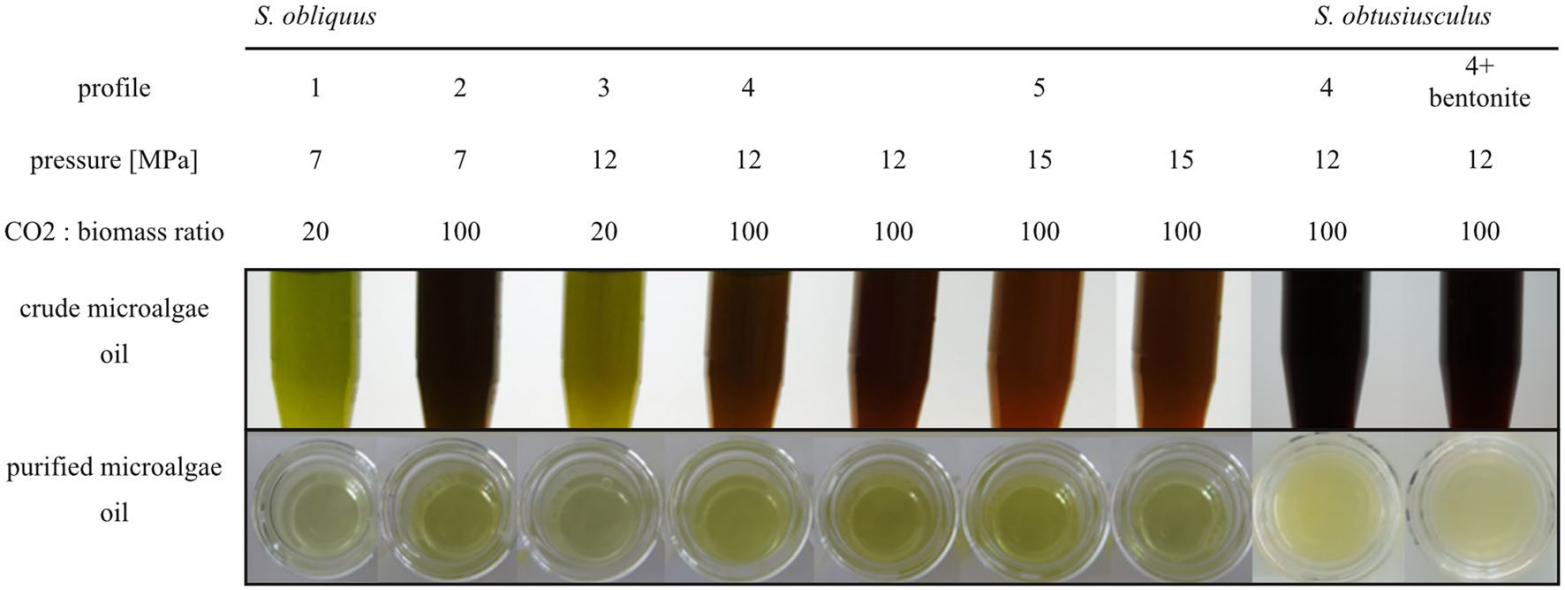
Microalgae oils obtained from different extraction profiles before (crude) and after (purified) the processing with a bentonite

#### Ion analysis of microalgae lipid extracts

For a further comparison of the microalgae extracts before and after the processing with a bentonite, the amounts of selected ions were analyzed (Table 6).

**Table 6:** Ion analysis of microalgae lipid fractions obtained from different extraction profiles, before (crude) and after (cleaned) a bentonite processing (in mg/kg)

	*S. obliquus*														*S. obtusiusculus*	
Profile	1		2		3		4				5				4	
Pressure (MPa)	7		7		12		12		12		15		15		12	
CO_2_: biomass ratio	20		100		20		100		100		100		100		100	
	Crude	Cleaned	Crude	Cleaned	Crude	Cleaned	Crude	cleaned	crude	Cleaned	Crude	Cleaned	Crude	Cleaned	Crude	Cleaned
Al	0.9	<0.5	1.9	<0.5	5	<0.5	5.5	<0.5	5.4	<0.5	3.8	<0.5	3.3	<0.5	0.8	<0.5
Ca	6.9	1.3	22	2.1	19	0.9	31	0.5	33	1.2	19	<0.5	15	0.8	1.4	1.9
Cu	<0.5	2	<0.5	0.9	<0.5	0.9	<0.5	1	<0.5	<0.5	<0.5	<0.5	<0.5	<0.5	0.7	<0.5
Fe	<0.5	<0.5	<0.5	<0.5	<0.5	<0.5	<0.5	<0.5	<0.5	<0.5	<0.5	<0.5	<0.5	<0.5	0.9	<0.5
K	<0.5	1.9	<0.5	2.8	<0.5	0.8	<0.5	0.8	<0.5	0.7	<0.5	<0.5	1	<0.5	0.7	<0.5
Mg	1	<0.5	<0.5	0.7	<0.5	<0.5	<0.5	<0.5	<0.5	<0.5	<0.5	<0.5	<0.5	<0.5	<0.5	<0.5
Na	<5	10	<5	9	<5	5	<5	6	<5	6	<5	<5	<5	<5	10	<5
P	21	<4	18	<4	35	<4	34	<4	30	<4	31	<4	30	<4	<4	<4

The results in Table 6 show no significant differences between the applied extraction profiles. All analyzed elements occur in very low amounts (e.g., maximum 33 mg/kg for calcium or 35 mg/kg for phosphor). After treating the samples with the bentonite, the amounts of calcium and phosphor strongly decrease whereas the amount of potassium, sodium and copper slightly increase.

### SCCO_2_ with online bentonite processing

Based on the results shown above an online processing of microalgae biomass with bentonite was tested. The bentonite was implemented directly into the extraction vessel and the resulting extracts were compared with extracts obtained from standard extractions. The total lipid yield of the extraction with implemented bentonite was with 6.7% (w/w) in the range of the standard extractions. The color and viscosity was also similar to the extracts obtained with the extraction profile 4 obtained before the subsequent bentonite bleaching.

The comparison of the microalgae extracts gained from standard extraction and online bentonite processing shows minor differences in the FA composition. The amount of polyunsaturated fatty acids (PUFA) is slightly increased, but the accuracy of the measurements is lower (Table 7).

**Table 7:** Major fatty acid (FA) components in microalgae extracts from standard extractions (standard) compared to extractions with online bentonite processing (bentonite), expressed as percentage of the total lipid extract (mean ± standard deviation)

	*S. obtusiusculus*		
Profile	4	4	
	Standard	Bentonite	
C14	0.16 ± 0.00	0.21 ± 0.04	
C16	3.25 ± 0.05	4.69 ± 1.00	
C16:1 cis ω7	0.45 ± 0.01	0.55 ± 0.07	
C16:1 ω9	0.41 ± 0.03	1.07 ± 0.44	
C16:2 ω6	0.71 ± 0.01	1.09 ± 0.29	
C16:3 ω3	1.13 ± 0.02	1.66 ± 0.42	
C16:4 ω3	3.91 ± 0.07	7.07 ± 2.58	
C18	0.17 ± 0.01	0.18 ± 0.01	
C18:1 ω9	4.09 ± 0.03	4.98 ± 0.69	
C18:1 ω7	0.79 ± 0.00	0.96 ± 0.16	
C18:2 ω6	6.93 ± 0.07	8.51 ± 1.24	
C18:3 ω3	20.62 ± 0.29	24.3 ± 3.11	
C18:4 ω3	0.88 ± 0.02	1.67 ± 0.68	
C20	ND	ND	
C20:1 ω9	ND	ND	
FA yield	43.52 ± 0.52	56.94 ± 10.66	

All extractions were carried out at 20 °C

*FA yield* percentage of FAs from total lipids, *ND* not detectable

The comparison of the ion analysis between microalgae oils gained from standard extraction and online bentonite processing is shown in Table 8. The results show similar amounts of the analyzed elements in both samples, before and after the subsequent cleaning procedure. The ion concentration does not significantly differ between the analyzed oils from standard extraction and online bentonite processing.

**Table 8:** Ion analysis of microalgae lipid fractions obtained from standard extraction and online bentonite processing of *S. obtusiusculus*, before (crude) and after (cleaned) a final bentonite processing (in mg/kg)

	*S. obtusiusculus*			
Profile	4		4	
	Standard		Bentonite	
	Crude	Cleaned	Crude	Cleaned
Al	0.8	<0.5	1.2	<0.5
Ca	1.4	1.9	1.7	0.7
Cu	0.7	<0.5	0.6	<0.5
Fe	0.9	<0.5	1.2	<0.5
K	0.7	<0.5	0.6	<0.5
Mg	<0.5	<0.5	0.6	<0.5
Na	10	<5	<5	<5
P	<4	<4	<4	8.5

## Discussion

### Effect of pressure and temperature on extraction yield

The different extraction yields shown in the Tables 1 and 2 all result from the same biomass processed, but small differences, especially in the soxhlet-yields after the extraction, are detectable. These differences are a result of a marginal loss of lipids on the inner surfaces of the processing equipment. The combination of this low loss and slightly differing batch sizes leads to differences in the lipid yields in a low percentage range. This effect can be reduced by increasing batch sizes and continuous extractions in the same plant.

The dark extracts obtained from the first extractions under harsh conditions suggest that the combination of high temperature and pressure lead to a denaturation of some algae cell components. The different coloring of the extracts after extractions under milder conditions can be explained by the varying concentrations of chlorophyll (green) and carotenoids (reddish-brown) inside the samples. This hypothesizes an increasing extraction efficiency of carotenoids by an increased CO_2_ to biomass ratio.

The maximum lipid yields obtained with the applied extraction protocol (Tables 1, 2, 3) are relatively low, compared to other published data for *S. obliquus* [11]. This can be explained by the single use of carbon dioxide as solvent for the extraction. The technique of using polar co-solvents to increase the extraction yields is commonly found in the literature [13]. An implementation of this technique into existing industrial extraction facilities is, with respect to the current ATEX directives (2014/34/EU), extremely complicated and expensive. In addition, it is important to mention that the processed microalgae have not been cultivated under optimal conditions for lipid production. Under nitrogen starving conditions, an increase of intracellular lipids of 200% is reported for *S. obtusiusqulus* [14]. Furthermore, there is a big difference between the sample sizes processed in this work (650–1300 g dried biomass) and the data published so far. Since most of the data published result from sample sizes of 0.5–1 g of dried biomass in laboratory-scale plants, it is unclear if these results are reproducible at a technically relevant scale.

### Processing of microalgae oils with bentonites

For any downstream application of microalgae oils a high quality and purity is essential. High concentrations of trace elements, for example, are not only problematic for chemical catalysts [15], but can also influence the activity of biological catalysts. Therefore, a concentration of trace elements higher than 5 ppm should not be exceeded. The scalable cleaning procedure applied to the microalgae lipid fractions, using bentonites, showed excellent results (Table 5). Besides the visible removal of the coloring compounds inside the microalgae extracts, the processing with bentonite also reduces the concentration of trace elements (Table 6) under the level of 5 ppm.

### SCCO_2_ with online bentonite processing

The online bleaching of the microalgae extracts by implementing the bentonite directly into the extraction vessel had only a minor visual effect on the extracts (Table 5), although the bentonite showed a strong green coloring after the extraction. However, the online processing had an effect on the subsequent cleaning step. The extracts obtained from the online bleaching showed a lower viscosity and enhanced flow behaviour on the cleaning column. The analysis of the corresponding FA profiles (Table 7) showed that the major components were similar to standard extractions. Nevertheless, further optimization steps for the online bleaching, e.g., differing amounts of bentonite inside the extraction vessel, need to be tested to reach higher purities for microalgae extracts.

## Conclusion

In this study, we optimized the process for SCCO_2_ extraction of algae biomass under industrially relevant conditions for the first time. All reactions were carried out in an industrial pilot plant (operated under ATEX directives) to maximize the lipid extraction performance from dried microalgae biomass and to obtain a high quality product with minimized concentrations of organic and inorganic contaminants. The mild extraction conditions (12 MPa pressure, a temperature of 20 C and a CO_2_ to biomass ratio of 100) applied in the optimized process resulted in a high lipid recovery of up to 92% w/w of total lipids (containing up to 59% w/w of PUFAs) from the microalgae biomass. To utilize the crude microalgae lipid fractions for following downstream applications, a scalable purification procedure, using the bentonite type industrial adsorber Tonsil 510®, was successfully integrated into the process. The microalgae lipid fractions generated by this procedure comply with all qualifications as a feedstock stream to in end-product formulations particularly for biolubricant and nutraceutical applications.

## Abbreviations

SCCO_2_: Supercritical carbon dioxide
PUFA: Polyunsaturated fatty acid
FA: Fatty acid
MPa: Mega pascal
FAME: Fatty acid methyl ester
